# Endocrine-Disrupting Chemicals and Disease Endpoints

**DOI:** 10.3390/ijms24065342

**Published:** 2023-03-10

**Authors:** Changhwan Ahn, Eui-Bae Jeung

**Affiliations:** 1 Laboratory of Veterinary Physiology, College of Veterinary Medicine, Jeju National University, Jeju 63243, Republic of Korea; 2 Laboratory of Veterinary Biochemistry and Molecular Biology, College of Veterinary Medicine, Chungbuk National University, Cheongju 28644, Republic of Korea

**Keywords:** endocrine system, endocrine disrupting chemical, endocrine-related diseases

## Abstract

Endocrine-disrupting chemicals (EDCs) have significant impacts on biological systems, and have been shown to interfere with physiological systems, especially by disrupting the hormone balance. During the last few decades, EDCs have been shown to affect reproductive, neurological, and metabolic development and function and even stimulate tumor growth. EDC exposure during development can disrupt normal development patterns and alter susceptibility to disease. Many chemicals have endocrine-disrupting properties, including bisphenol A, organochlorines, polybrominated flame retardants, alkylphenols, and phthalates. These compounds have gradually been elucidated as risk factors for many diseases, such as reproductive, neural, and metabolic diseases and cancers. Endocrine disruption has been spread to wildlife and species that are connected to the food chains. Dietary uptake represents an important source of EDC exposure. Although EDCs represent a significant public health concern, the relationship and specific mechanism between EDCs and diseases remain unclear. This review focuses on the disease-EDC relationship and the disease endpoints associated with endocrine disruption for a better understanding of the relationship between EDCs-disease and elucidates the development of new prevention/treatment opportunities and screening methods.

## 1. Introduction

Various endocrine-disrupting chemicals (EDCs) are found in the environment. These EDCs affect hormone synthesis or receptor binding by altering the hormone homeostasis of the endocrine system [1]. EDCs can cause reproductive, developmental, and sexual behavior dysfunctions, leading to detrimental results in animals and human beings. Most EDCs from natural or synthetic sources have structures similar to those of endogenous steroid hormones, including estradiol(E2) or androgen. Hence, they tend to interfere with the actions of steroid hormones by binding to the corresponding hormone receptors [2].

Endocrine disruption by EDCs can occur by altering the normal hormone levels, inhibiting or stimulating the production of hormones, or changing the way hormones travel throughout the body, thus affecting the functions of these hormones [3]. EDCs were initially thought to exert their actions solely through nuclear hormone receptors (NRs), including estrogen receptors [ERs, i.e., Bisphenol A(BPA) and dioxins] [4], androgen receptors (ARs, i.e., pesticides, phthalates, plasticizers, polyhalogenated compounds), progesterone receptors (PR), thyroid receptors [TRs, i.e., BPA, dioxins, perchlorates, furans], and retinoid receptors (i.e., Organotins, BPA) [3,5]. On the other hand, recent evidence has shown that the mechanisms through which EDCs act are much broader than originally recognized [4]. Indeed, studies have shown that in addition to altering nuclear receptor signaling, EDCs can act through the nonsteroid receptors, transcriptional coactivators, enzymatic pathways involved in steroid biosynthesis and metabolism, and numerous other mechanisms that converge upon endocrine and reproductive systems [5,6]. Other less well-known mechanisms of action of EDCs include the direct effects on genes [7]. Several important principles demonstrate how environmental exposures increase the risks of adult disease [8]. First, chemical exposure can have both tissue-specific and time-specific consequences on growth and development. Second, the pathophysiology may be manifested in a disease that might otherwise not have occurred and disease progression may have variable latent periods. Finally, the effects of environmental chemical exposures can be transgenerational, thereby affecting future generations.

Unlike the traditional mechanism of action of EDCs, recently, EDCs are regarded as a trigger for endoplasmic reticulum stress; however, the basal molecular mechanisms regarding EDCs and the endoplasmic reticulum are still lacking. Nevertheless, there is several evidence that some EDCs may have apoptotic effects on various cells in the body in relation to inducing endoplasmic reticulum stress depending on the concentration of exposure [9,10,11,12,13,14,15]. Endocrine-disrupting chemicals such as bisphenol A, alkylphenols, dioxins, perchlorates, Furans, and pesticides that enter the aquatic ecosystem not only interferes with aquatic organisms but also with terrestrial and aerial animals linked directly or indirectly with water through food chains or other ecological interactions [16]. The presence of chemicals exhibiting endocrine-disrupting properties has significantly increased in the ecosystem [5]. Therefore, there is growing concern about their potential ability to induce/exacerbate diseases in relation to EDCs. Indeed, due to their wide use and direct link to adverse human health concerns, the Endocrine Society published a scientific statement in 2009 indicating that endocrine disruptors pose a “significant concern for public health” [5]. Possible diseases in relation to endocrine-disrupting chemical have been listed. By providing a better understanding of diseases related to EDC exposure and health risks, this review intends to give information for risk management.

## 2. Brief Properties of EDCs

### 2.1. Complex Mechanisms of EDCs

Some EDCs act via several mechanisms and have mixed steroidal properties. For example, an EDC may have both estrogenic and anti-androgenic or have estrogenic and progesterone properties [5]. Moreover, the metabolite of EDCs could have different actions compared to its original structure. For instance, the estrogen agonist, Dichlorodiphenyltrichloroethane (DDT), is metabolized into the androgen antagonist Dichlorodiphenyldichloroethylene (DDE) [17]. EDCs can also act through genomic and non-genomic mechanisms. Genomic responses are delayed and require several hours to become established [18]. In addition to genetic modulation, non-genomic responses occur rapidly, often within minutes of exposure. EDCs act by modulating the endogenous steroid hormone metabolism, nuclear receptor coactivators (NCOAs), and proteasome-targeted degradation of endogenous hormones. The mechanisms of EDCs involve divergent pathways including estrogenic, androgenic, thyroid, peroxisome proliferator-activated receptor γ (PPARγ), retinoid, and other nuclear receptors. Endocrine-disrupting chemicals are thought to act primarily through NRs including ERs, ARs, PRs, TRs, and others [5]. Thus, endocrine disruptors act via NR, nonnuclear steroid hormone receptors (e.g., membrane ERs), nonsteroid receptors (e.g., neurotransmitter receptors such as the serotonin receptor, dopamine receptor, norepinephrine receptor), orphan receptors [e.g., aryl hydrocarbon receptor (AhR)—an orphan receptor], enzymatic pathways involved in steroid biosynthesis and/or metabolism, and numerous other mechanisms [5]. 

EDCs such as BPA, zearalenone (Zea), and nonylphenol (NP) also had relatively high binding affinities for G protein-coupled estrogen receptors (GPER) [19,20,21]. GPER-dependent signaling pathway activated by estrogens. Following nontraditional estrogen actions mediated through GPER are a key mechanism to disruption by a variety of environmental estrogens [22]. In contrast, EDCs such as polychlorinated biphenyls (PCBs) and dioxin bind with relatively high affinity to the AhR [23,24]. It regulates the transcription of a large group of dioxin-responsive genes and results in a reduction in cytosolic estrogen levels [25]. EDCs exert their toxic effects by interfering with hormonal production, secretion, and action that affect the growth and development of reproductive tissues [25]. These exogenous chemicals interfere with the binding of hormones to their receptors such as ERs and ARs which can result in an agonistic or antagonistic effect [5]. For example, an organochlorine pesticide (methoxychlor) has been reported to cause estrogenic action by binding to estrogen receptor α (ERα) and estrogen receptor β (ERβ) subtypes [26]. In addition to receptor interference, EDCs can also interfere with enzyme action involved in steroidogenesis. Phthalates and such plasticizers exert anti-androgenic activity by disrupting steroidogenesis in the H295R assay [27]. It has been reported that some EDCs inhibit 5-α reductase that converts testosterone to dihydrotestosterone [28,29]. Thus, EDCs can affect hormone receptor expression. It has been reported that BPA alters the epigenome and causes the malregulation of steroid receptors [30].

Some EDCs such as BPA and alkylphenols exert endoplasmic reticulum (ER) stress. The exact mechanism of how EDCs cause endoplasmic reticulum stress still needs to be studied. On the other hand, endoplasmic reticulum stress markers must be significantly increased in order to indicate EDC exposure. Endoplasmic reticulum stress activates a signaling network called the unfolded protein response (UPR) to restore endoplasmic reticulum homeostasis. However, under prolonged and severe endoplasmic reticulum stress by EDCs, the UPR can become cytotoxic [14,31].

### 2.2. Transgenerational Effects

Mutations or subtle modifications of gene expression induce the transgenerational effects of EDCs. It is still unclear how EDC exposure during early development leads to phenotypic changes that manifest as diseases much later in life or even in the next generation [32]. On the other hand, increasing evidence suggests a central role for epigenetic mechanisms in the transgenerational effects of EDCs [5]. In recent years, several experimental studies and some evidence from epidemiology have shown that EDCs induce epigenetic changes [33]. Epigenetic modifications are the “heritable and reversible modifications of chromatin, resulting in an adjustment of its activity without changing the underlying DNA sequence, such as histone modification and non-coding RNA” [32]. Epigenetic alterations of the germline can include DNA methylation, histone modifications, and noncoding RNAs [34,35]. Epigenetic alterations can be transmitted through the germline to the unexposed generation to cause effects on subsequent generations and cause transgenerational phenomena [34,35]. By DNA methylation, DNA methyltransferases add a methyl group to the cytosine base, and methylation is usually associated with the repression of transcription. Histone modification involves altering the chromatin structure; it also takes a part in the regulation of gene expression [36]. Lastly, noncoding RNAs are involved in chromatin function and can modulate gene expression with gene silencing. Known EDCs that affect male and female reproduction include phthalates, BPA, pesticides, and environmental contaminants, including polychlorinated biphenyls (PCBs) and 2,3,7,8-tetrachlorodibenzo-p-dioxin (TCDD) [35,37,38,39,40]. Those EDCs which effects the germline showed negative effects on male and female fertility in a transgenerational manner. In animal studies with females individuals, ancestral (primary) EDC exposure can alter transgenerational litter size and anogenital distance, cause early puberty, disrupt estrous cyclicity, alter follicle numbers, decrease fertility, cause early reproductive aging, increase cysts in ovaries, alter sex steroid hormone levels, and cause adenomyosis. Furthermore, in males, ancestral EDC exposure can alter transgenerational anogenital distance, cause testes disease, cause early puberty, decrease fertility, decrease sperm count and motility, alter sperm morphology, and alter sex steroid hormone levels [35,37,38,39,40,41,42].

## 3. Disease Endpoints in Relation to EDCs

### 3.1. Reproductive Disorders

Clinical and experimental data suggest that the prenatal and prepubertal effects of EDCs manifest in male and female children in the form of altered pubertal timing [43,44]. In particular, EDCs that have estrogenic properties, such as alkylphenol, BPA, and phthalates, could induce precocious puberty [45]. In a female pubertal assay in a rat model on the effects of parabens, a significant decrease in serum estradiol and thyroxine concentration, a significant delay in the date of vaginal opening, disruption in the length of the estrous cycle, and a decrease of corpora lutea were observed resulting from long-term exposure to parabens. These results showed less estrogenic activity than estradiol, which can suppress hormonal responsiveness and disrupt the morphology of the reproductive target tissues [46,47]. Many EDCs are known as xeno-estrogen which bind to the estrogen receptor (ER) with an affinity approximately 1000-fold lower than that of estrogen. These EDCs appear to induce tissue-specific estrogenic responses as an ER agonist or antagonist, resulting in dysregulation of ERα-dependent transcriptional signaling pathways [48].

**Figure 1:**
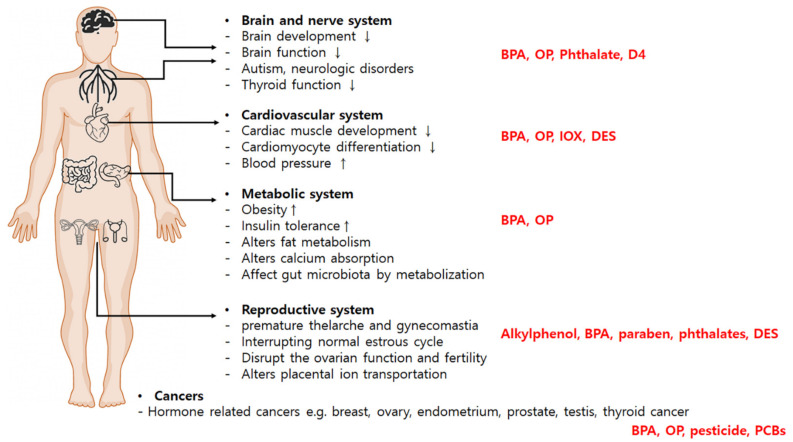
Schematic overview of endpoints of diseases affected by Endocrine Disrupting Chemicals (EDCs). Exposure to EDCs contributes significantly to the onset and progression of organ development and disorders such as reproductive, metabolic, neurologic, cardiovascular disease, and cancers; these EDCs include Bisphenol A (BPA), octylphenol (OP), octamethylcyclotetrasiloxane (D4), ioxynil (IOX), diethylstilbestrol (DES), polychlorinated biphenyls (PCBs).

Prenatal exposure to EDCs, particularly diethylstilbestrol (DES) and BPA, is associated with human female reproductive malformations, as well as cysts, adenomas, and carcinomas in the reproductive tissues. Clinical and experimental data also indicate that exposure to EDCs affects fertility by interfering with multiple processes, including folliculogenesis, steroidogenesis, ovulation, fertilization, and gestation, which means EDCs disrupt ovarian function and fertility (Figure 1). Numerous studies have suggested that exposure to EDCs may increase the incidence of uterine fibroids by promoting estrogen-dependent hyperplasia of the myometrium, endometriosis, premature ovarian failure, and polycystic ovary syndrome (PCOS) [49,50,51,52]. In addition, exposure to EDCs during early gestation disrupts intrauterine implantation and uterine reception, leading to implantation failure [53].

Exposure of pregnant women to estrogenic or mixtures of EDCs that interfere with the male hormone action (e.g., anti-androgenic EDCs) increases the risk of cryptorchidism in their sons, causing reduced semen quality and an increased risk of subfertility and testicular cancer in adult life [54]. Data from vertebrate experimental animals and clinical studies demonstrate that early exposure to multiple classes of EDCs may result in male reproductive disorders [55]. In the context of male reproductive disorders, EDCs have been linked to disrupted reproductive functions, manifesting as reduced semen quality and infertility as well as altered fetal development, which are displayed as urogenital tract abnormalities, including hypospadias and cryptorchidism [5,56]. Animal and experimental data prove the effect of EDCs on the male reproduction system. Hypospadias, cryptorchidism, and oligospermia have been reported in men exposed to EDCs [5,57]. Laboratory experiments with rats and epidemiological studies strongly suggest that the co-occurrence of cryptorchidism, hypospadias, testis germ cell cancer, and impaired semen quality results from reduced androgen action during fetal development, causing testicular dysgenesis syndrome [58,59]. Using rat models, previous studies showed that a wide range of anti-androgenic and estrogenic EDCs can cause reproductive dysfunction [60].

Di-phthalate and genistein alter gene expression in the testis [61]. Clinical data indicate that postnatal exposure to dichlorodiphenyltrichloroethane (DDT), BPA, and phthalates is associated with significant decreases in sperm quality via genomic/non-genomic estrogen signaling and anti-androgenic activity [62,63,64]. Testicular dysgenesis syndrome (TDS) occurs due to the inhibition of androgen action on fetal development preceding a Sertoli and Leydig cell dysfunction that may result from direct or epigenetic effects [65].

Exposure to EDCs can occur through the ingestion of food, dust, and water, inhalation of gases and particles in the air, and biological transfer across the placenta. The placenta is another route of EDC exposure. In the process of exposure, EDCs can affect placental transportation. Cation transporters, such as transient receptor potential cation channels in subfamily V, member 6 (TRPV6), plasma membrane calcium-transporting ATPase 1 (PMCA1), solute carrier family 31, member 1 (SLC31A1), ATPase Copper Transporting Alpha (ATP7A), Hephaestin (HEPH) genes and proteins are altered by octylphenol (OP) and BPA [66]. The fetus relies on the mother for ionic transportation. Therefore, pregnant women should avoid exposure to cation-channel-disrupting EDCs.

### 3.2. Metabolic Disorders

The global epidemic of metabolic disorders has been attributed to endogenous factors, such as genetics, diet, lifestyles, and aging [67]. On the other hand, there is also increasing evidence of the relationship between metabolic disorders and EDCs. EDCs can promote metabolic changes that lead to metabolic disorders and may play an important role in the global epidemic of metabolic diseases [68]. Some studies have reported a correlation between EDCs and various factors affecting metabolic diseases, such as body fat and obesity (Figure 1) [69]. Studies suggest that phthalate exposure could result in approximately 54,000 cases of obesity in older European women [70]. Clinical research has also demonstrated that EDC exposures are associated with obesity [71,72]. 

Other clinical studies have shown that BPA exposure is linked to the risk of insulin resistance and type 2 diabetes mellitus (T2D) in women of reproductive age, including pregnant women [73,74]. Epidemiologic studies have also shown that an elevation of urinary phthalate metabolites in women of all ages increases the incidence of diabetes mellitus [75]. In an insulin-dependent diabetes mellitus mouse model, the disruption of calcium homeostasis by BPA- and OP-induced endoplasmic reticulum stress led to insulin resistance [76]. BPA and OP treatments lead to the survival of pancreatic β cells against streptozotocin, but the serum glucose regulation is not properly regulated despite the increased insulin level [14,76]. This may convert insulin-dependent diabetes patients to insulin-independent patients. In another pregnant rat model, the fat metabolism altered by OP may have affected the nutrition balance during pregnancy and cause metabolism-related diseases [77]. OP down-regulated adipogenesis-associated gene expression, which was mediated by the regulation of their transcription factors. Therefore, the decrease in adipogenic enzyme expression reduced fat deposition in adipocytes and body weight in pregnant rats. These metabolic changes may affect the maintenance of pregnancy and the transportation of nutrients to the fetus. Therefore, an OP-induced nutritional imbalance can cause severe metabolic diseases, such as ketosis, marasmus, and diabetes mellitus in pregnant individuals [77].

EDCs can be ingested via the intestines; EDCs also affect various intestinal cell types. EDCs affect calcium transport via the intestinal epithelia by regulating calcium transportation-related genes [78]. Moreover, EDCs are also metabolized by the gut microbiota, which may alter their toxicodynamics [79]. Therefore, studies coupled with EDC–microbiota interactions will help determine the effects of EDCs.

EDCs can also promote nonalcoholic fatty liver disease (NAFLD) by increasing free fatty acid (FFA) uptake, increasing lipogenesis, decreasing triglyceride exportation via very low-density lipoprotein(VLDL), and/or decreasing FFA β-oxidation [80]. Relevant to the NAFLD, several of these EDCs including BPA, tributyl tin(TBT), PCBs, phthalates, perfluorooctanoic acid (PFOA), and perfluorooctanesulfonic acid (PFOS) are classified as obesogen [81]. EDCs exert their activity as endocrine disruptors via their ability to activate genomic and non-genomic pathways of NRs and thus act as NR agonists or antagonists. The liver expresses an extensive repertoire of NRs that have important roles in hepatic lipid metabolism.

### 3.3. Neurologic Disorders

There are concerns regarding the potential effects of EDCs on brain function, particularly in relation to psychiatric, cognitive, and behavioral disorders over the past few decades [82]. EDCs may act upon NRs that are expressed in hypothalamic or pituitary cells, thereby exerting feedback effects [83]. Steroid hormone receptors are expressed abundantly in the hypothalamus and other brain areas that control neuroendocrine functions [84].

Epidemiologic data suggest that exposure to EDC may result in a range of neurological developmental defects and functional deficiencies [85]. In other cohort studies, prenatal exposure to EDCs was associated with cognitive difficulties and abnormal social behavior [86,87]. EDC exposure has been associated with learning abilities and linked to lower intelligence quotients (IQs) [88,89]. On the other hand, epidemiological studies have yielded conflicting results. Data from mother-child pairs in the Mount Sinai Children’s Environmental Health Study with a multi-ethnic urban population indicated that exposure to prenatal phthalate was associated with childhood social impairment [90].

Animal model studies showed results that proves the relationship between EDCs and neurologic disorders. Cyclic siloxane octamethylcyclotetrasiloxane (D4) exerts estrogen activity that affects the cell cycle progression of neuronal progenitor cells during neurodevelopment, which may be associated with cognitive deficits in their offspring [85]. Another study showed that perinatal exposure to OP disrupts brain development and behavior in mice by decreasing the length of axons and dendrites and increasing the number of primary and secondary dendrites, resulting in a reduction of neuronal progenitor proliferation in the brains of offspring mice [91]. During the SARS-CoV-2 pandemic, the usage of triclosan (TCS) was widely considered [92]. TCS is one of the biocides which are products that kill or inactivate the growth of harmful microorganisms including bacteria, fungi, and viruses [93]. However, some other potential alternatives such as nonorganic antibiotics and organic or natural biocides have been proposed in place of TCS due to some of the reported side effects [94]. TCS can impair dendrite and axon growth by reducing the average length and numbers of axons and dendrites, resulting in a cognition dysfunction and impairments in sociability, social novelty preference, and anxiety-like behavior, which are symptoms of neurodevelopment disorders [95].

Thyroid hormones are essential for normal development. Slight fluctuations in maternal or fetal hormones are associated with impaired neurodevelopment in the offspring [96]. EDCs affect thyroid function in several ways, including hormone biosynthesis, transport, metabolism, and TR activity [97]. Thus, clinical and animal studies have suggested that EDC exposure could impair thyroid signaling during development and be associated with neurological deficits [98,99,100]. Clinical studies have proven that exposure to polybrominated diphenyl ethers (PBDEs) is associated with hypothyroidism [101]. Another brominated flame retardant, 2,4,6-tribromophenol (TBP), can disrupt thyroid hormone homeostasis and the presence of TBP influenced thyroid actions as regulators of gene expression. The risk of these agents interfering with neural development by disrupting the thyroid hormone signaling axis needs to be considered.

### 3.4. Cardiovascular Development

The endocrine system homeostasis is important for normal development. In particular, steroid hormones, such as E2 and progesterone (P4), influence the calcium signaling of the cardiac muscle in early embryo development [102]. Estradiol and P4 are endogenous steroid hormones with various important functions in the body, including regulation of the menstrual cycle, maintaining pregnancy, and embryogenesis. Similarly, progesterone reportedly contributes to the cardiac repolarization processes (Figure 1) [103]. 

In an in vitro study, exposure to OP and BPA led to inhibitory effects on calcium signaling in cardiomyocyte differentiation [102]. The study showed that OP and BPA have a similar structure to endogenous steroid hormones, such as progesterone and estrogen, and OP and BPA act like progesterone to inhibit and disrupt the cardiomyocyte differentiation of mouse embryonic stem cells (mESCs) [102]. In a similar study, exposure to ioxynil (IOX) and DES during the embryonic stage led to a disruption of cardiovascular development.

A cohort study to evaluate the effect of prenatal exposure to BPA on the blood pressure of the child showed that children born to women with elevated maternal BPA concentrations during the second trimester of pregnancy display significantly higher diastolic blood pressure [104]. Meta-analysis of 33 epidemiological studies showed that exposure to BPA was associated with hypertension and coronary artery disease [71].

### 3.5. Cancers

Hormone-related cancers or hormone-sensitive cancers, such as breast, endometrium, ovary, prostate, testis, and thyroid cancer, share a unique mechanism. Endogenous and exogenous hormones drive cell proliferation and affect the accumulation of random genetic errors [105]. EDCs work as exogenous hormones, promoting cell proliferation and exaggerating tumor progression. 

In breast cancer, endogenous sex steroid hormones are one of the risk factors. Excess estrogen exposure in utero was associated with an increased incidence of breast cancer [106]. Surprisingly, the impact of EDCs with estrogenic activities has increased the breast cancer risk. EDC exposure in the intrauterine environment can affect the risk of developing breast cancer later in life. A longitudinal clinical study showed that women exposed to DDT during pregnancy increased their daughter’s risk of breast cancer, independent of the maternal history of breast cancer [107]. Other statistics indicate that for more than 50,000 women in the United States and Puerto Rico, who were sisters of women with breast cancer, the use of hair dyes (containing EDCs) and straighteners was associated with an increased risk of breast cancer [108]. An animal model supported the possible link between EDCs and breast cancer. In a xenografted mouse model, TCS and OP help breast cancer progression associated with an ER-mediated signaling pathway [109]. Moreover, a recent study showed that EDCs also introduce epithelial-mesenchymal transition (EMT) in relation to ER, making cancer cells migrate into other organs [110].

The incidence of testicular germ cell cancer (TGCC) has been continuously rising in many countries, including Europe and the U.S. [111]. EDCs that can mimic or disturb steroid hormone actions may disrupt testicular development and adversely affect reproductive health at birth, during puberty, and in adulthood. Hypospadias, cryptorchidism, and poor semen quality are elements of TDS that may be considered risk factors for TGCC [65]. Studies on the TGCC risk in men exposed to PCBs in utero, such as DDE (a metabolite of DDT) or benzenes, have been confirmed [112,113]. However, the effective concentrations of EDC exposure on testicular cancer are controversial. In the testicular tumor environmental and endocrine determinants study, a case-control study of 754 case subjects, the serum DDE levels correlated with the risk of seminomatous and non-seminomatous TGCCs [114]. This result suggests that TGCC is initiated in early life. Moreover, exposure to these persistent organic pesticides during fetal life or via breastfeeding may increase the risk of TGCC in young men. On the other hand, two smaller case-control studies did not find this association [112,113]. Animal models are still not used for TGCCs but will need to be considered in the near future.

Prostate cancer is the third leading cause of cancer deaths in American men [115]. Large epidemiologic studies of approximately 90,000 participants showed a direct and highly significant correlation between the incidence of prostate cancer and exposure to methyl bromide (a fungicide) and six different pesticides [116,117]. In addition to agricultural chemicals, arsenic, cadmium, and PCBs have been linked to prostate cancer [118]. The precise mechanisms through which these chemicals induce the carcinogenic process in the prostate remain to be resolved, although one shared property is their estrogenic activities. An in vivo human prostate stem cell model showed that EDCs with estrogenic activities initiate and promote prostatic carcinogenesis in an androgen-supported environment. These findings suggested that tissue stem cells may be direct EDC targets. Moreover, developmental or transient adult exposure may cause life-long reprogramming [119,120]. EDC exposure was also found to cause epigenetic changes within several genes involved in prostate cancer initiation and growth [121,122]. These changes involved increased methylation and decreased gene expression. Furthermore, chronically elevated estrogen in men resulting from allelic variants in aromatase has been associated with an elevated risk of prostate cancer [123]. In one report, oral contraceptive use by mothers during pregnancy was positively correlated with prostate cancer risk and mortality in male offspring [124]. In one animal model, exposure to estrogen or estrogenic EDCs resulted in developmental estrogenization/estrogen imprinting in the rodents for prostate cancer [65].

## 4. Cohort Studies: Impact of EDCs on Disease Endpoints

Cohort studies are one of the most powerful tools researchers have to understand human health. They involve following groups of participants for long periods of time and examining trends in the data. These trends can be important for identifying causes and risk factors for diseases. Nevertheless, all the participants in the epidemiological study are not in the same circumstances and characteristics. Therefore, these traits could produce an inconsistent result in each study.

The reproductive system is complex and requires normal development and function, including the ovary, uterus, vagina, testis, and anterior pituitary. EDCs have the potential to interfere with the structure and functions of reproductive organs by adversely affecting their development and functions. Urinary levels of phthalates/BPA are associated with pubertal gynecomastia, premature thelarche, and precocious puberty in girls [125,126]. The effects on the menstrual cycle by EDCs are also examined by epidemiology studies; a cross-sectional study of women residing in agricultural areas showed an association between atrazine exposure and increased menstrual irregularity [127]. The Effect of EDCs on fertility was also studied with an epidemiological method; in a study of 137 women undergoing in vitro fertilization, an association between increased urinary BPA concentrations and increased implantation failure was found [128]. Moreover, assessing exposure to a mixture of 66 persistent EDCs, females’ preconception concentrations of polybrominated diphenyl ether and cadmium were positively associated with incident hCG pregnancy loss in a cohort of couples from the general population trying for pregnancy [129]. Similar to interference with the female reproductive system, a series of reviews found consistent significant associations between pesticide exposure and reduced sperm concentration and motility across decades [130,131].

A number of cross-sectional epidemiological studies associate the metabolite of the pesticide DDT levels with obesity, diabetes mellitus, and cardiovascular diseases in humans [97]. Metabolic diseases such as T2D and obesity have interlinked and overlapping pathologies; there is sufficient evidence to conclude that some EDCs act as obesogens and others act as diabetogens [97]. In 2002, Baillie and Hamilton announced the obesogenic action of toxic chemicals such as perfluoroalkyl and polyfluoroalkyl substances, based on the parallel increase of pollutants and the incidence of obesity [132]. Since 2008, many epidemiological studies have proven the association between EDCs and metabolic disease [17,133,134,135,136,137]. Prenatal exposure to phthalates increases body mass index and risk of being overweight [138]. Another epidemiological research shows the associations in women who have prenatal DES exposure with coronary artery disease and myocardial infarction which appear to be independent of established cardiovascular disease risk factors [139]. Long-term exposure to BPA, PCBs, and phthalate can influence cardiovascular health in humans. A possible synergistic effect may exist between the homologs [140].

The experimental animal study consistently shows that the structure and function of the brain’s neuroendocrine function can be altered by developmental exposures to EDCs [91,95,134]. In humans, epidemiological data support associations between higher exposures to phthalate and decreased IQ, increased neurodevelopmental problems, and other neurocognitive outcomes [134,141,142,143].

The incidence of hormone-sensitive cancers of the breast, uterus, ovary, and testis has been hypothesized to increase these types of cancers due to various EDCs in the environment [134]. Research on the effects of EDCs on cancer progression has proceeded over the past few years, with epidemiological and rodent studies supporting links between them. The majority of EDCs are highly persistent in the environment and bio-accumulative, therefore it is essential to assess the long-term impacts of EDC exposures (Table 1). In the meta-analysis, EDCs have a potential link to endocrine-related cancer progression such as ovarian, breast, and prostate cancers [144,145,146]. Estrogenic EDCs such as phthalate, BPA, dioxin, and PCBs exposure have a link to breast cancer risk [147,148,149,150]. Similar to animal studies, pesticides (i.e., vinclozolin and DDT, DDE) induce a high risk of incident for the TGCC [151,152].

The epidemiology studies such as prospective and retrospective cohort studies have been performed. Nevertheless, trying to interpret diverse and sometimes contradictory results can lead to confusion and the impression that there is still controversy about whether or not EDCs have biological effects on humans. It might be the exposure dosage or individual characteristics. To better understand the EDCs’ adverse effects, a well-designed cohort study should be considered.

## 5. Conclusions

Humans are exposed to thousands of chemicals during their lifetime through the air they breathe, the food they eat, and the water they drink. A significant number of these chemicals disrupt the endocrine system. Endogenous hormones play essential roles in cell proliferation, differentiation, tissue development, and maintaining cell function in organs. The similar structure of EDCs means that they can also bind with endogenous hormone receptors and block the signals. Furthermore, EDCs may trigger disorders in various organs, such as reproductive abnormalities, metabolic diseases, endocrine dysfunction, and cancers. 

EDCs include a vast group of chemicals from natural (i.e., phytoestrogens) and man-made materials (i.e., DES and BPA) and are found in a variety of consumer products (e.g., pharmaceuticals and personal care products, cleaning products, antimicrobials, food preservatives, and phthalates). The view that EDCs are significantly affecting human health is of great concern. More data is needed to expand the list of tissues affected by EDCs, and more effort is needed to identify and classify the consequent diseases and dysfunctions in humans and animal models. Better information on how and when EDCs act is needed to reduce exposures during development and prevent diseases. The review identified the need for improved testing methods for EDCs, the importance of reducing exposure and vulnerability to disease, and suggested the necessity for methods evaluating evidence to be applied to improve human and wildlife health.

## Figures and Tables

**Table 1:** Summaries of the EDCs–disease relationship on body.

System	Organ	Disease	Chemicals	Ref
Reproductive system	Uterus	Uterine fibroid	Bisphenols, phthalates, pesticides (DDT, DDE, endosulfans, DES, etc.)	[53,153]
	Endometrium	Endometriosis	Bisphenols, phthalates, pesticides (chlorpyrifos, HCB), dioxin, PCBs	[154,155,156]
	Ovary	Infertility, subfertility	DES, phthalates, bisphenols, parabens, heavy metals, dioxin, PCBs, pesticides (DDT, DDE), triclosan	[5,157]
		Irregular reproductive cycles, Early menopause, PCOS	Phthalates, bisphenols, dioxins, PCBs, pesticides (DDT, DDE), parabens, DES, OP, NP, triclosan	[50,51,52,158]
	Breast	Breast cancer	Bisphenols, phytoestrogens, DES, TCDD, PCBs, DDT, DDE, pesticides (vinclozolin)	[108,159,160]
	Testis	Infertility, subfertility	Phthalates, PCBs, Pesticides, Pesticides (vinclozolin, ethylene dibromide)	[35,161,162]
		Cryptorchidism	Bisphenols, Phthalates, dioxins, PCBs, pesticides (vinclozolin), parabens, DES	[5,58,157]
		Hypospadias	Phthalates, DES, progestin, loratadine, clomiphene, pesticides (vinclozolin, DDT, atrazine)	[163,164,165]
Metabolic system	Pancreas	Pancreatic β cell damage,	Bisphenols, DDT, OP, nonylphenol, rodenticide (pyrinuron)	[14,70,75,166]
	Intestine	Type II diabetes	Bisphenols, DDT, octylphenol, NP, phthalates	[14,166,167]
		Changes in Gut microbiota	Dioxins, pesticides, pyrethroids, PCBs, flame retardants, triclosan	[79]
	Adipose tissue	Alters fat metabolism, hypertrophy or hyperplasia of adipocytes	Bisphenols, DES, PCBs, Tributyltin, OP	[168,169]
Nervous system	Brain	psychiatric, cognitive, and behavioral disorders, Autism Spectrum Disorders	Bisphenols, Diethylstilbestrol, phthalates, pesticides, octamethylcyclotetrasiloxane, OP, triclosan	[91,95]
Neuroendocrine system	Thyroid	Decrease in hormone biosynthesis, transport, metabolism, and thyroid hormone receptor (TR) activity	Bisphenols, PBDEs, TBP, PCBs, phthalates, perchlorate	[101,170,171]
Cardiovascular system	Heart	Abnormal cardiovascular development, interrupted calcium signaling, increasing the probability of cardiovascular disease,	Bisphenols, OP, ioxynil, DES, TCDD, DDT	[71,102]
-	Cancers	Hormone-related cancers or hormone-sensitive cancers, such as breast, endometrium, ovary, prostate, testis, and thyroid cancer Parkinson’s disease	Bisphenol, DDT, triclosan, OP, TCDD, DES, phthalates (Breast cancer) Bisphenols, DDT, PCBs, phthalates (Uterine cancer) Bisphenols, Herbicides (chlorotriazine), DES, TCDD (Ovarian cancer) DDE, benzenes, (Testicular cancer) Bisphenols, DES, pesticides (endosulfans, malathion, vinclozolin), Agricultural chemicals, Dioxin, arsenic, cadmium, PCBs (Prostate cancer)	[65,107,108,110,112,113,115,116,117,118,119,121,122,123,124,159,160,172,173,174,175,176,177,178,179,180,181]

## Abbreviations

Endocrine disrupting chemicals (EDCs), estradiol (E2), nuclear hormone receptors (NRs), estrogen receptors (ERs), androgen receptors (ARs), progesterone receptors (PRs), thyroid receptors (TRs), bisphenol A (BPA), dichlorodiphenyltrichloroethane (DDT), dichlorodiphenyldichloroethylene (DDE), nuclear receptor coactivators (NCOAs), peroxisome proliferator activated receptor γ (PPARγ), aryl hydrocarbon receptor (AhR), zearalenone (Zea), nonylphenol (NP), G protein-coupled estrogen receptor (GPER), poly chlorinated biphenyls (PCBs), estrogen receptor α (ERα), estrogen receptor β (ERβ), unfolded protein response (UPR), 2,3,7,8-tetrachlorodibenzo-p-dioxin (TCDD), diethylstilbestrol (DES), polycystic ovary syndrome (PCOS), dichlorodiphenyltrichloroethane (DDT), testicular dysgenesis syndrome (TDS), transient receptor potential cation channels in subfamily V, member 6 (TRPV6), plasma membrane calcium-transporting ATPase 1 (PMCA1), solute carrier family 31, member 1 (SLC31A1), ATPase Copper Transporting Alpha (ATP7A), Hephaestin (HEPH), Octylphenol (OP), type 2 diabetes mellitus (T2D), nonalcoholic fatty liver disease (NAFLD), free fatty acid (FFA), very low density lipoprotein (VLDL), tributyl tin(TBT), perfluorooctanoic acid (PFOA), perfluorooctanesulfonic acid(PFOS), Intelligence quotients (IQs), triclosan (TCS), polybrominated diphenyl ethers (PBDEs), 2,4,6-tribromophenol (TBP), progesterone (P4), mouse embryonic stem cells (mESC), ioxynil (IOX), testicular germ cell cancer (TGCC).
